# The Tortoise Beetles of an Atlantic Forest Mountain Range: Host Plants and Temporal Distribution

**DOI:** 10.1007/s13744-026-01411-9

**Published:** 2026-07-21

**Authors:** Felipe Capoccia, Lílian Andrade, Marianna V. P. Simões, Fernando A. Frieiro-Costa, João Vasconcellos-Neto

**Affiliations:** 1 Programa de Pós-Graduação em Ecologia, Instituto de Biologia, Univ of Campinas (Unicamp), Campinas, São Paulo Brazil; 2 Lab de Interações Animais e Plantas, Depto de Biologia Animal, Instituto de Biologia, Univ of Campinas (Unicamp), Campinas, São Paulo Brazil; 3 Senckenberg Research Institute and Natural History Museum, Frankfurt, Germany; 4 Lab de Zoologia, Zoology Sector, Univ Federal de Lavras (UFLA), Lavras, Minas Gerais Brazil

**Keywords:** Community ecology, Cassidinae, Insect–plant interaction, Seasonality

## Abstract

Tortoise beetles (Cassidinae s. str., Chrysomelidae, Coleoptera) compose a highly diverse group of insects, with most species found in Neotropical regions, such as the Brazilian Atlantic Forest, one of the most important hotspots in the world. Neotropical cassidines are known to typically display strong host specialization and distinct temporal patterns; however, host plants of most species are still unknown and very few studies have quantitatively investigated their temporal distribution. Here, we present a comprehensive list of tortoise beetle species for Serra do Japi, an Atlantic Forest mountain range, by comparing and combining data from 26 months of field work with data from previous samplings done in the same area. By collecting richness and abundance of adults for 12 consecutive months, as well as monthly mean temperature and precipitation, we also tested the correlation between assemblage and climatic factors. Our final list is composed of 71 cassidines species, with 11 new distribution records for the state of São Paulo and new host plant records for 36 of these species. We also show that the adults of this tortoise beetle assemblage present a seasonal pattern, similar to that of subtropical cassidines, which is significantly influenced by temperature variation, but not by precipitation. Our results highlight the importance of Serra do Japi as a conservation unit and contribute to a better understanding of several ecological aspects of Cassidinae *s. str.*

**Supplementary Information:**

The online version contains supplementary material available at 10.1007/s13744-026-01411-9.

## Introduction

The subfamily Cassidinae Gyllenhal (Chrysomelidae), comprising over 6300 species and 34 tribes globally, stands as the second largest subfamily among leaf beetles (Borowiec and Świętojańska 2014; Leocádio et al. 2020). With greatest diversity in tropical regions, Cassidinae are prominent for their extraordinary morphological diversity, specialized plant associations in the form of herbivory, and a range of social behaviors from solitary to subsocial (Gomes et al. 2012; Leocádio et al. 2020; Borowiec and Świętojańska 2024) . Within Cassidinae, two ecologically distinct lineages are recognized: the predominantly endophagous leaf-mining beetles (formerly treated as Hispinae), largely associated with monocotyledonous plants; and the exophages tortoise beetles (Cassidinae sensu stricto), that feed externally on dicotyledonous hosts.

Cassidinae *s. str.* are distinguished by their rounded body and the expanded margins of the pronotum and elytral, features that give rise to their common name, “tortoise beetles” (Borowiec 1999). Brazil, the largest country in the Neotropics, harbors 1173 species and more than 565 endemic species, representing 41.2% of the world’s fauna (Borowiec 1999; Sekerka and Simões 2025). The Brazilian Atlantic Forest is recognized as one of the world’s most important biodiversity hotspots due to its exceptional species richness and endemism (Myers et al. 2000). However, because relatively few faunal surveys have been conducted so far, knowledge of Cassidine diversity in this biome remains limited (Flinte et al. 2008; Simões and Monné 2011; Fernandes and Linzmeier 2012; Frieiro-Costa et al. 2012; Gomes et al. 2021).

Tortoise beetles typically display strong host specialization, feeding on the surface of the leaves of one to few plant species in all life stages (mono- to oligophagy), and maintain close and often prolonged relationships with their host plants (Bernays and Chapman 1994; Windsor et al. 1992; Buzzi 1994). Consequently, their spatial and temporal distribution is strongly related to availability and phenology of host plants (Maulik 1948; Chaboo 2007). However, despite being one of the most intensively studied chrysomelid subfamilies, host plant information remains surprisingly limited: only about 63% of all tortoise beetle genera have host records, with most published data identifying beetles only to genus, and host plants only to family level (Jolivet and Hawkeswood 1995; Borowiec 1999). As a result, detailed and reliable species-level host plant–insect interactions remain very incomplete, leaving major gaps in our understanding of feeding specificity, ecology, and distribution patterns (Chaboo 2007; Gomes et al. 2021).

Neotropical species of Cassidinae *s. str.* generally exhibit distinct temporal patterns, with many reproducing—defined here as the presence of mating adults and/or immature stages—only during the warmest and most humid seasons (Wolda 1978, 1987, 1988; Flinte et al. 2011). In Brazil, tropical cassidines species tend to be found throughout the year and seem to be only weakly influenced by climatic factors, while subtropical species commonly overwinter in diapause, exhibiting stronger patterns and a closer association with climate variables (Nogueira-de-Sá et al. 2004). On tropical mountains, however, climate has an even greater effect on community structure, and seasonality similar to that observed in subtropical regions may emerge (Flinte et al. 2011). Unfortunately, few studies have conducted quantitative analyses to investigate their temporal distribution patterns in these environments (Nummelin and Borowiec 1992; Flinte et al. 2011, 2015; Gomes et al. 2020).

The Serra do Japi, located in São Paulo, Brazil, is one of the largest remnants of Atlantic Forest, occupying an area of 35,000 ha. The area forms a small mountain range, with altitudes ranging from 700 to 1300 m above sea level and is situated within an ecotone, which results in high heterogeneity of soil types and microclimatic conditions (Pinto 1992; Santoro and Machado 1992). These factors create an array of microhabitats, allowing a great diversity of native fauna and flora with different requirements to occur within this region, which makes Serra do Japi an important refuge for the biodiversity of distinct forest formations of the Atlantic Forest (Leitão-Filho 1992; Rodrigues and Sheperd 1992). Consequently, this region represents an ideal natural laboratory for investigating the diversity, host associations, and seasonal dynamics of tortoise beetles in montane Atlantic Forest ecosystems.

Considering the substantial gaps in the current knowledge of Neotropical tortoise beetles, particularly in montane Atlantic Forest systems, such as Serra do Japi, more research is needed to verify their diversity, temporal and geographical distribution, and host plant associations (Frieiro-Costa et al. 2012; Gomes et al. 2021). Here, we present a comprehensive list of tortoise beetle species and their host plants for an Atlantic Forest mountain range by combining our data with those from Frieiro-Costa et al. (2012) (1985–1995) and Thiago Marinho Alvarenga (unpublished host plant data from 2013). Using abundance and richness data of adult beetles collected over 12 consecutive months, we also analyze seasonality and discuss how climatic factors might influence their temporal distribution. We hypothesize that this species assemblage shows a seasonal pattern similar to that of subtropical species given the climate of this tropical mountain range. We also predict that the temporal distribution of adult beetles is positively correlated with both monthly variation of temperature and precipitation.

## Material and methods

### Study area and sampling

#### Study area

**Fig. 1:**
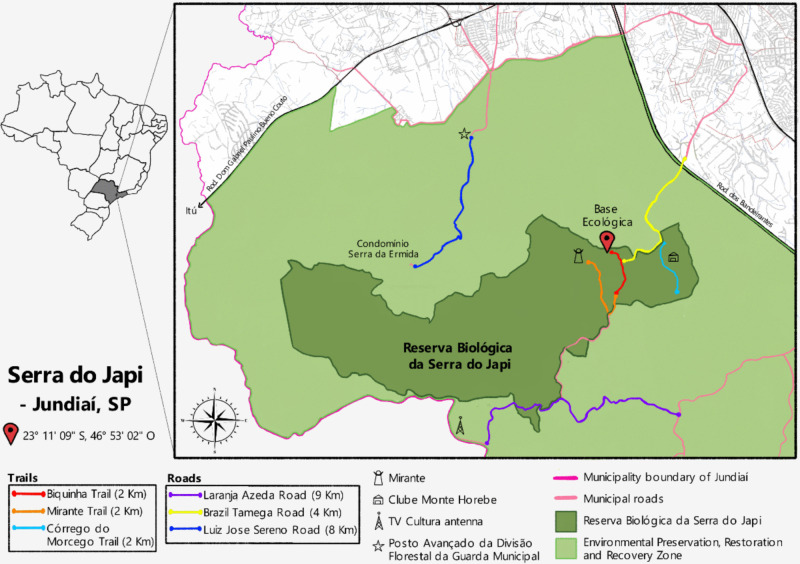
Map of the area of Serra do Japi where samplings were done. Trails and roads where beetles and their host plants were sampled are indicated by different colors. The map also indicates some of the main reference points to find the sampling locations in the conservation unit. Illustration made in Krita, adapted from the maps available at https://serradojapi.jundiai.sp.gov.br/institucional/mapas/mapas/

This study was conducted at Serra do Japi, Jundiaí, São Paulo, Brazil (23°11′S; 46°52′W), under the care of Fundação Serra do Japi (Fig. 1). The conservation unit at Jundiaí occupies an area of 91.40 km^2^ of Atlantic Forest, corresponding to the largest portion of Serra do Japi (47.67% of the entire area). The climate of the region is strongly seasonal, with rainy summers and dry winters, classified as Cwa type in the classification of Köppen (Monsoon-influenced Humid Subtropical Climate) (Pinto 1992). Due to the altitude, the temperatures throughout the year are moderate, with mean variation of 15.7 to 19.2 °C. The hottest month is January, when temperatures vary from 18.4 to 22.2 °C; while in July, the coldest month, the temperatures vary from 11.8 to 15.3 °C (Pinto 1992; Frieiro-Costa et al. 2012). The rainy season goes from October to March, with its peak between December and January when precipitation surpasses 200 mm per month. The dry season goes from May to August when rain is scarce, varying from 30 to 60 mm monthly (Morellato 1992; Pinto 1992).

#### Sampling

Surveys were done in six different sites of the conservation unit, three trails, and three unpaved roads (Fig. 1). Each site is located at different altitudes and shows different conditions of sun exposure. The access to the trails and one of the roads is restricted and requires authorization; the other two roads are public but very isolated, mainly used by researchers and guards. The areas are affected by human intervention mainly through the clear off in the border, guided visits to the trails and occasional cars on the roads. The sampling was done monthly on each of these locations for a total of 26 months, specifically from March to August of 2023 and from January of 2024 to August of 2025 (SISBIO authorization 93020-1 and 93020-2).

Beetles were collected by two people actively searching on native vegetation on both edges of the trails and roads for six hours each (26 visits: 156 h on each location; 156 visits: 936 h in total). Distances searched varied on each location according to how many potential host plants were found, but, in total, around 1404 km were searched within 26 months of field work. All native vegetation found within 2 m from the road and trail edges, and at a height up to 3 m from the ground were visually inspected for tortoise beetles or feeding marks, which is one of the best indicators of their presence (Liu et al. 2019; Gomes et al. 2021). Tortoise beetles’ feeding marks vary a lot, with some species leaving entire holes in the leaves while others only scrap their surface (Chaboo 2007; Gomes et al. 2021). Since some tortoise beetles hide on the underside of leaves while feeding (Flinte et al. 2015; Gomes et al. 2021), all leaves with any kind of feeding marks were carefully turned using tweezers to avoid scaring potential beetles.

Active search is the most common way to collect Cassidinae *s. str.* since traps are not effective and do not allow reliable associations with host plants (Fernandes and Linzmeier 2012; Gomes et al. 2021). Adult beetles were collected and maintained for a week in containers with leaves from the plants on which they were found. The interaction was considered confirmed only when the beetle was observed feeding on the leaves or feeding traces were observed after some days. To avoid interfering with abundance data, beetles were collected only if the species’ host plant had not been confirmed yet or if it was a beetle species not previously recorded; otherwise, we only noted how many specimens of each species were found on each known host plant. Immatures were also collected and kept under the same care until they became adults, allowing the species identification. All host plants were marked and had their coordinates recorded, allowing subsequent collection once flowers and fruits were available for accurate species-level identification.

Vouchers of beetles were identified by Felipe Capoccia (Instituto de Biologia, University of Campinas, Brazil) and Marianna Simões (Senckenberg Research Institute and Natural History Museum Frankfurt, Germany) and deposited in the zoological collection of the Museu de Diversidade Biológica da UNICAMP (MDBio) (Online Resource 1). Plant vouchers were identified by plant taxonomist Pedro Quinellato Dantas, with assistance of Luisa Suzuki and Felipe Capoccia (Instituto de Biologia, University of Campinas, Brazil), and are deposited in the herbarium of the MDBio. The growth habit of the host plants for each of the tortoise beetle species was also recorded (i.e., vines, herbs, shrubs or trees) following Flora e Funga do Brasil (2025).

### List of species and host plants

Previously, Frieiro-Costa et al. (2012) published a list of Cassidinae *s. str.* for Serra do Japi using occurrence data sampled between 1988 and 1995, but with no records of their host plants. This list was based on occasional, non-systematic collections, without standardized sampling effort, or abundance data. We also had access to unpublished host plant data collected by Dr. Thiago Marinho Alvarenga (Instituto de Biologia, University of Campinas, Brazil) in 2013 as part of his PhD Thesis (Vouchers deposited in Laboratótio de Interações Animais e Plantas, Instituto de Biologia, University of Campinas, Brazil). Data from all periods was sampled through active search at the same six locations of the conservation unit and were used to complement the list of tortoise beetles presented here.

To verify the geographic distribution of beetle species and their known host plants, we consulted the literature data and the catalogs (Borowiec and Świętojańska 2024 and Sekerka and Simões 2025). The species list follows the taxonomic status accepted in Borowiec and Świętojańska (2024). For each species, we provide information on new records based on the examined material, the known geographic distribution and host plant associations.

### Temporal distribution data and climatic variables

To describe the temporal distribution of tortoise beetles in this community, we considered only abundance and richness data collected from September 2024 to August 2025, corresponding to part of the sampling described above. Because Brazil was under the influence of El Nino during the first semester of 2024, we restricted our analyses to data collected after this period in order to avoid potential climatic bias. Abundance data were recorded exclusively for the adults, as larvae of different species are morphologically very similar and could not be reliably distinguished in the field.

Climatic variables, including average monthly temperature and monthly accumulated precipitation, were obtained from the Jundiaí ETEC meteorological station for the study period (data available at http://www.ciiagro.org.br/janeladofruticultor/index.php). The station is located about 7 km away from the sampled areas.

### Data analysis

To evaluate sampling completeness, we applied individual-based interpolation (rarefaction) and extrapolation using a multinomial model implemented in R package iNEXT (Chao et al. 2014; R Core Team 2024; Hsieh et al. 2025). These analyses estimated the expected richness under increased sampling effort and assessed the representativeness of our dataset for the tortoise beetle assemblage of Serra do Japi. In addition, we calculated the nonparametric richness estimators Chao1 and ACE with the vegan package in R to estimate the potential richness of the assemblage based on the observed abundance data (O’Hara RB 2005; R Core Team 2024).

Seasonality in the assemblage was tested using circular statistics, applying Rayleigh uniformity in Oriana 4.02 software (Kovach Computing Services, UK). This analysis enabled identification of peaks in abundance and richness and assessment of whether their temporal distribution from September 2024 to August 2025 deviated from randomness.

To evaluate relationships between assemblage metrics and climatic drivers, we performed cross-correlation analyses between monthly assemblage responses (species richness and abundance) and climatic predictors (average temperature and accumulated precipitation) using a cross-correlation function (CCF) in R (R Core Team 2024). Correlations were evaluated across multiple temporal lags, allowing the detection of delayed biological responses to climatic variation.

## Results

### List of tortoise beetles and host plants

**Fig. 2:**
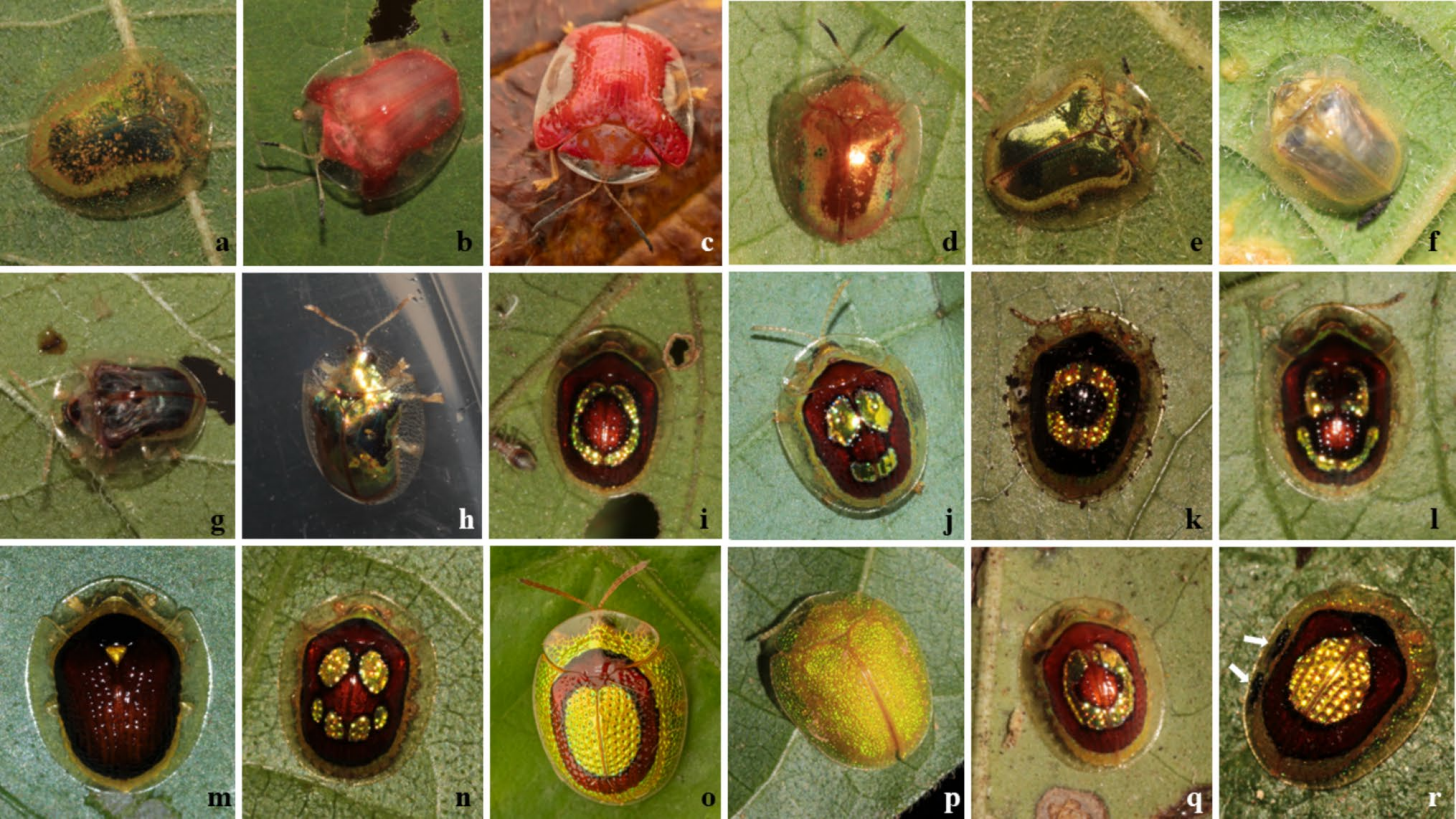
Tortoise beetles (Cassidinae *s. str.*) of the Serra do Japi, Jundiaí, Brazil found between 2023 and 2026. Tribe Cassidini: **a** *Charidotella* cf. *morio*, **b** *Charidotella* cf. *pudica*, **c** *Charidotella rubicunda* (credit: Pablo Levinsky), **d** and **e** *Charidotella sexpunctata*, **f** *Charidotella* sp. 1, **g** and **h** *Charidotella* sp. 2, **i** *Charidotis annularis*, **j** *Charidotis centromaculata*, **k** *Charidotis concentrica*, **l** *Charidotis contexta*, **m** *Charidotis furunculus*, **n** *Charidotis gemellata*, **o** *Charidotis mansueta* (credit: Pablo Levinsky), **p** *Charidotis marginella*, **q** *Charidotis pupillata*, **r** *Cteisella magica*. White arrows indicate parasitoid wasps

**Fig. 3:**
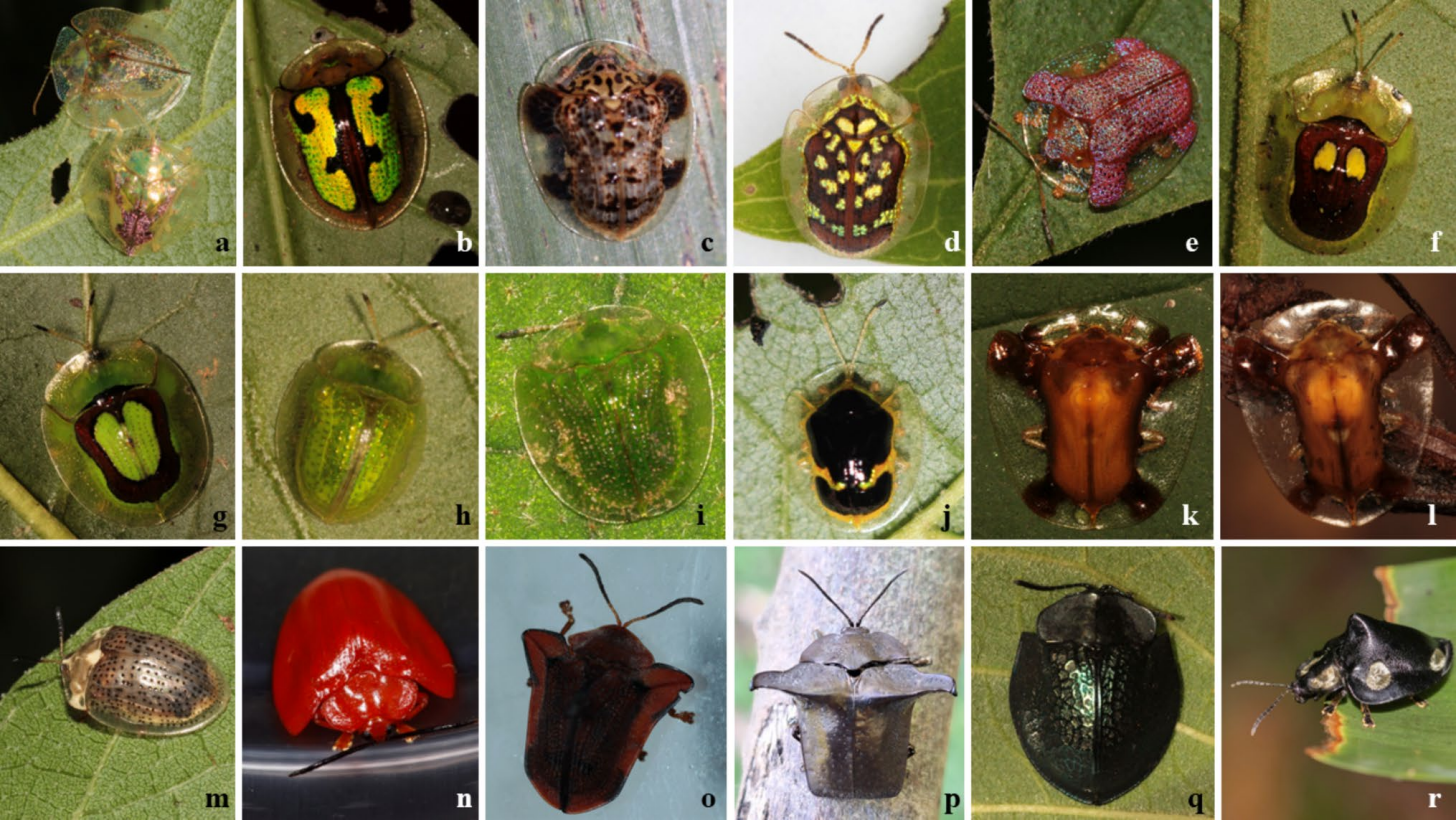
Tortoise beetles (Cassidinae *s. str.*) of the Serra do Japi, Jundiaí, Brazil found between 2023 and 2026. Tribe Cassidini: **a** *Coptocycla contemta*, **b** *Coptocycla fastidiosa*, **c** *Microctenochira aciculata*, **d** *Microctenochira brasiliensis* (credit: Yuri F. Messas), **e** *Microctenochira difficilis*, **f** *Plagiometriona clandestina*, **g** *Plagiometriona deyrollei*, **h** *Plagiometriona flavescens*, **i** *Plagiometriona punctatissima*, **j** *Ischnocodia succincta*, **k** *Syngambria andreae*, **l** *Syngambria bisinuata*; tribe Ischyrosonychini: **m** *Cistudinella obducta*; tribe Spilophorini: **n** *Calyptocephala nigricornis*; tribe Dorynotini: **o** *Dorynota monoceros*; tribe Omocerini: **p** *Omocerus truncatus* (credit: Pablo Levinsky); tribe Mesomphaliini: **q** *Cyrtonota thalassina*, **r** *Mesomphalia turrita* (credit: Pablo Levinsky)

**Fig. 4:**
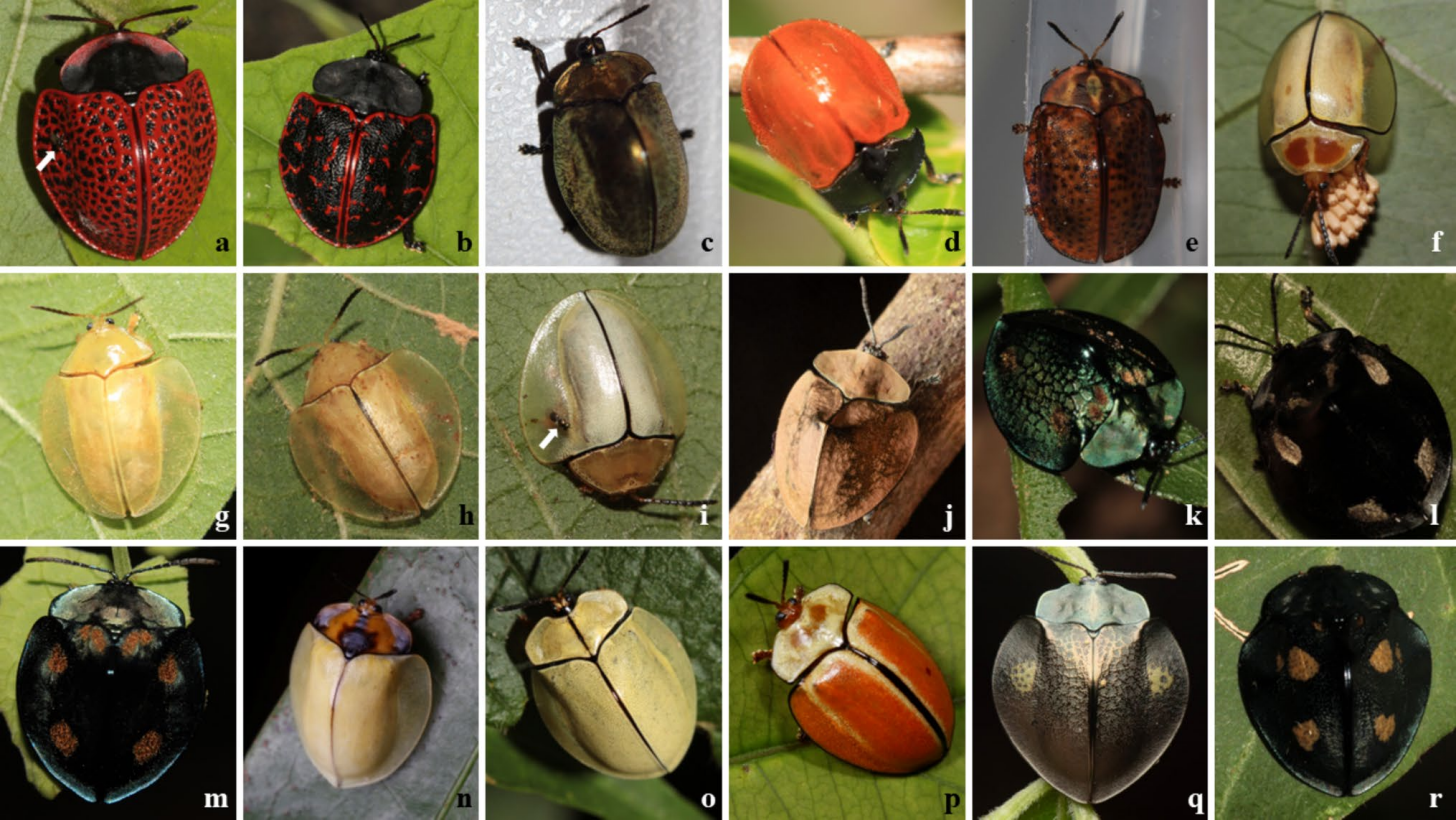
Tortoise beetles (Cassidinae *s. str.*) of the Serra do Japi, Jundiaí, Brazil found between 2023 and 2026. Tribe Mesomphaliini: **a** *Botanochara impressa*, **b** *Botanochara sigillata*, **c** *Chelymorpha clivosa*, **d** *Chelymorpha cribraria*, **e** *Chelymorpha inflata*, **f** *Omaspides brunneosignata*, **g** *Omaspides pallidipennis*, **h** *Omaspides trichroa*, **i** *Omaspides tricolorata*, **j** *Stolas antiqua* (credit: Pablo Levinsky), **k** *Stolas areolata*, **l** *Stolas brevicuspis*, **m** *Stolas chalybaea*, **n** *Stolas deleta* (credit: Yuri F. Messas), **o** *Stolas lineaticollis*, **p** *Stolas plagicollis* (credit: Yuri F. Messas), **q** *Stolas redtenbancheri* (credit: Pablo Levinsky), **r** *Stolas sexplagiata*. White arrows indicate parasitoid wasps

**Fig. 5:**
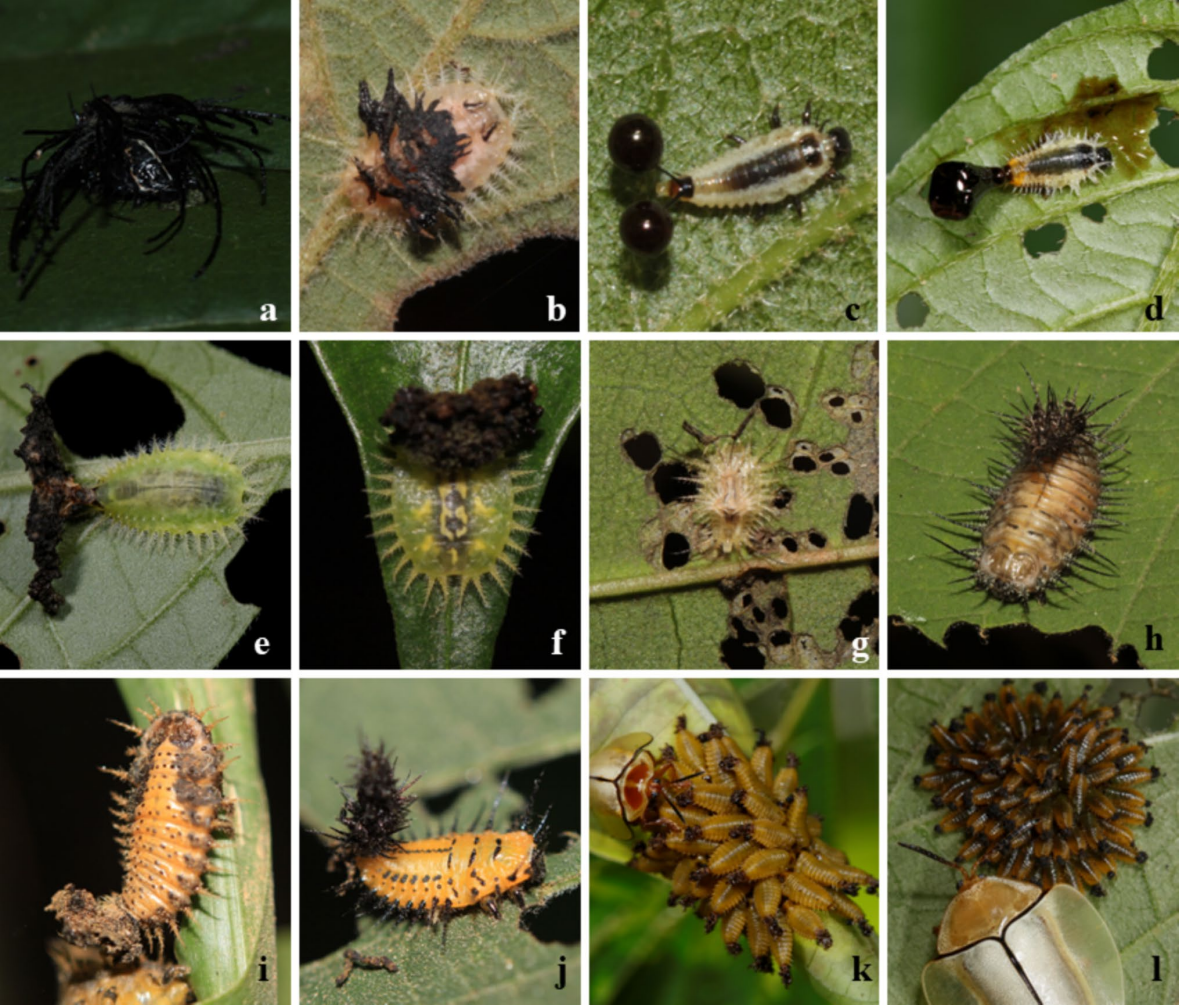
Immatures of the tortoise beetles (Cassidinae *s. str.*) of the Serra do Japi, Jundiaí, Brazil found between 2023 and 2026. Tribe Dorynotini: **a** *Dorynota monoceros*; tribe Cassidini: **b** *Charidotella* sp. 2, **c** *Coptocycla contemta*, **d** *Coptocycla fastidiosa*, **e** *Plagiometriona deyrollei*, **f** *Plagiometriona flavescens*, **g** *Syngambria bisinuata*; tribe Mesomphaliini: **h** *Botanochara sigillata*, **i** *Chelymorpha inflata*, **j** *Cyrtonota thalassina*, **k** *Omaspides brunneosignata*, **l** *Omaspides tricolorata*

**Fig. 6:**
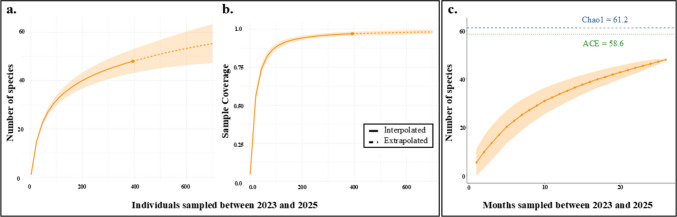
Individual-based interpolation (rarefaction) and extrapolation curves showing (**a**) number of Cassidinae *s. str.* species in relation to the number of individuals; **b** sample coverage in relation to number of individuals; **c** number of Cassidinae *s. str.* species in relation to the number of sampled months. Continuous line indicates the real sampling effort from 2023 to 2025 (interpolated data) at Serra do Japi, Jundiaí, Brazil. Dashed line indicates: **a** and (**b**) how many more species could be found in the area with 700 sampled specimens (extrapolated data) based on the multinomial model; **c** expected richness values according to the Chao1 (in blue) and ACE (in green) estimators

Our 26-month sampling effort yielded a total of 393 specimens, representing 48 species (Figs. 2, 3, 4, and 5). The multinomial model estimated that, under a 95% confidence interval, sampling an additional 309 more specimens (for a total of 700) would reveal up to 15 additional species (~ 63 species total; Fig. 6a), corresponding to a Sample Coverage (SC) of 0.9695 (Fig. 6b). Similarly, Chao1 and ACE estimators indicated expected richness values of 61.2 species and 58.6 species respectively based on the observed abundance data (Fig. 6c). Five additional species, *Omocerus truncatus* (Boheman), *Stolas deleta* (Boheman), *S. antiqua* (Sahlberg), *Charidotis centromaculata* (Boheman), and *Dorynota monoceros* (Germar), were found after our sampling period, between December 2025 and March 2026, and added to our final list, but were not included in any of our assemblage analysis*.* Compared with the previous survey conducted in the area (Frieiro-Costa et al. 2012), we recorded 30 newly documented species for Serra do Japi, while 18 species previously reported were not detected, and 23 species were shared between both datasets (Table 1).

**Table 1.**
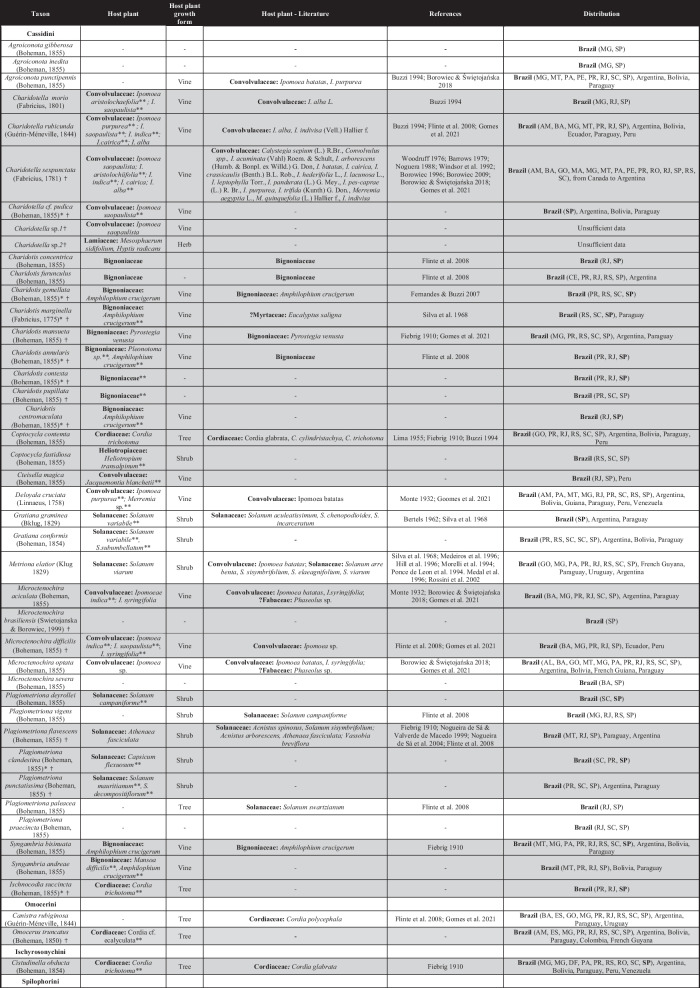

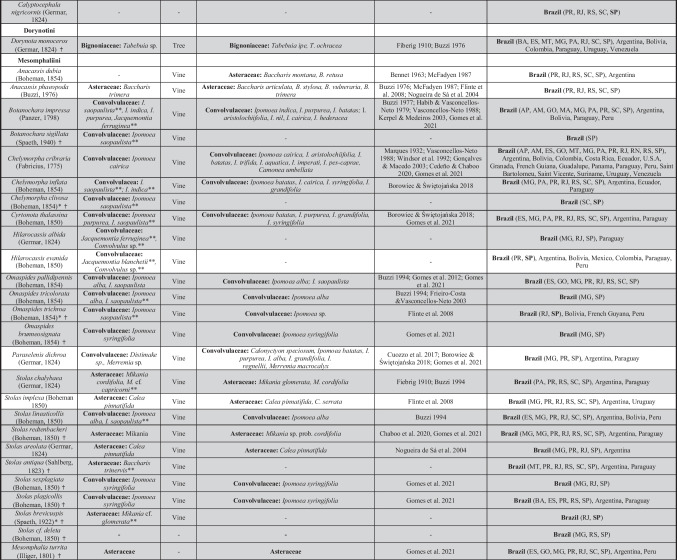
Species of tortoise beetles (Cassidinae *s. str*.) of Serra do Japi, Jundiaí, Brazil, and their respective host plants, including new host plant records (**). Distribution records, host plant growth form (Flora e Funga do Brasil 2025), host plants from literature, and the respective references are shown for each species. The new Cassidinae records for the state of São Paulo (*) were given following Borowiec and Świętojańska (2024) and Sekerka and Simões (2025). New records for Serra do Japi (✝) were given following Frieiro-Costa et al. (2012). Species in gray were found between 2023 and 2026

Immatures (larvae and pupae) were only found between December and April, when precipitation in the region is the highest. The sole exception was *Plagiometriona deyrollei* Boheman, for which immatures could already be found in October. Both adults and larvae feed on open leaves of their host plants, with adults generally feeding on the abaxial portion, while immatures were commonly found feeding on the adaxial portion of the leaves. Due to the frequent presence of parasitoids in both larvae and pupae, very few immatures reached imago and could be successfully identified. Only 12 species of the assemblage had their immatures confirmed, two of these species (*Omaspides tricolorata* Boheman and *O. brunneosignata* Boheman) present maternal care, while the others had solitary larvae (Fig. 5).

The combined dataset from our study and Frieiro-Costa et al. (2012) resulted in 71 species belonging to 26 genera and six tribes recorded for Serra do Japi (Table 1). Among these, 28 species are endemic to Brazil, and 11 species represent new records for the state of São Paulo, being *Charidotella pudica* Fabricius, *Charidotis gemellata* Boheman, *C. marginella* Fabricius, *C. annularis* Boheman, *C. contexta* Boheman, *C. centromaculata* Boheman, *Plagiometriona clandestina* Boheman, *Ischnocodia succincta* Boheman, *Chelymorpha clivosa* Boheman, *Omaspides trichroa* Boheman, and *Stolas brevicuspis* Spaeth. Apart from *C. pudica*, which is a new record for the country, all newly recorded species for the state were already known to occur in the southeast region of Brazil and only two of them (*C. marginella* and *O. trichroa*) are not endemic to the country. The tribes with the highest number of species are Cassidini (*n* = 40) and Mesomphaliini (*n* = 26), together accounting for 93% of all recorded species. The other four tribes, Omocerini (*n* = 2), Ischyrosonychini, Spilophorini, and Dorynotini (*n* = 1 each), represent 7% of the total. The richest genera are *Stolas* Bilberg 1820 (Mesomphaliini) (*n* = 10 species), *Charidotis* Boheman 1854 (Cassidini) (*n* = 9 species), and *Plagiometriona* Spaeth 1899 (Cassidini) (*n* = 7 species).

**Fig. 7:**
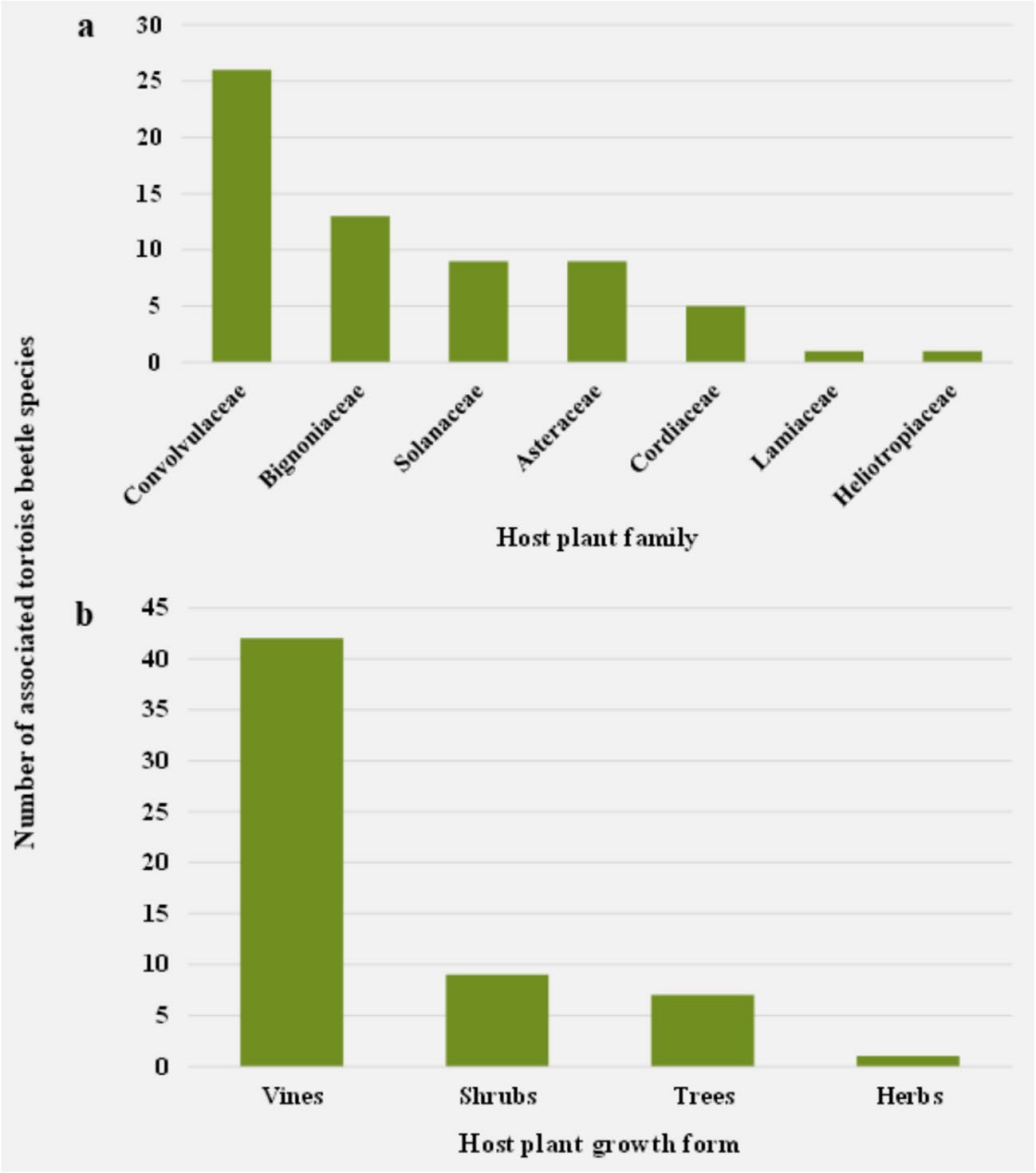
Richness of Cassidinae *s. str.* by host plant (**a**) family and (**b**) growth form. Serra do Japi, Jundiaí, Brazil

Host plants are now known for 64 of the 71 tortoise beetle species of the conservation unit, with 59 identified at species level (Table 1). We documented new host plant records for 36 tortoise beetle species, of which 19 represent the first host plant associations ever reported for these species. Most Cassidinae *s str.* species found feed on Convolvulaceae (*n* = 26, 40.6%), followed by Bignoniaceae (*n* = 13, 20.3%), Solanaceae and Asteraceae (*n* = 9, 14.1% each). Additionally, five species feed on Cordiaceae (7.8%) and only one species on Lamiaceae and Heliotropiaceae (3.1%) (Fig. 7a).

Regarding host plant growth form, tortoise beetle species of Serra do Japi are mostly associated with vines (*n* = 42, 71.2%), with fewer species recorded on shrubs (*n* = 9, 15.2%) and trees (*n* = 7, 11.9%), and only one species on herbs (1.7%) (Fig. 7b).

### Temporal distribution

**Fig. 8:**
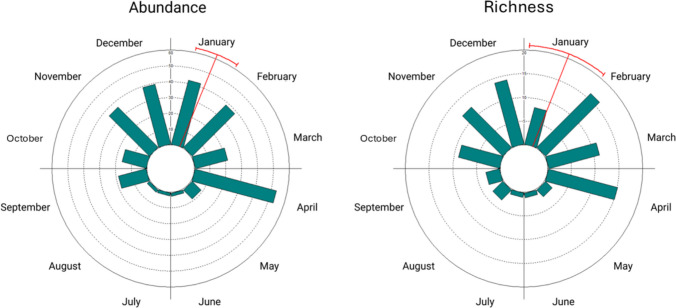
Circular graphs of monthly **a** abundance and **b** richness of adult tortoise beetles (Cassidinae *s. str.*) found between September 2024 and August 2025 at Serra do Japi, Jundiaí, Brazil. The red line indicates the mean vector (µ)

Two hundred seventy-seven tortoise beetle specimens were sampled between September 2024 and August 2025 (Table 2). The Rayleigh test resulted in a significant non-random distribution of abundance and richness values throughout the year (*p* < 0.001), indicating a pronounced seasonal pattern for this assemblage (Table 3). The highest values for both variables occurred between October and April, corresponding to the warm and rainy season, with a tendency to peak towards January (the median group) (Table 3 and Fig. 8). Between June and September, the coldest and driest months in the period, both variables were at their lowest. *Plagiometriona flavescens* (Boheman) was the only species found during the whole period (Table 2).

**Table 2.**
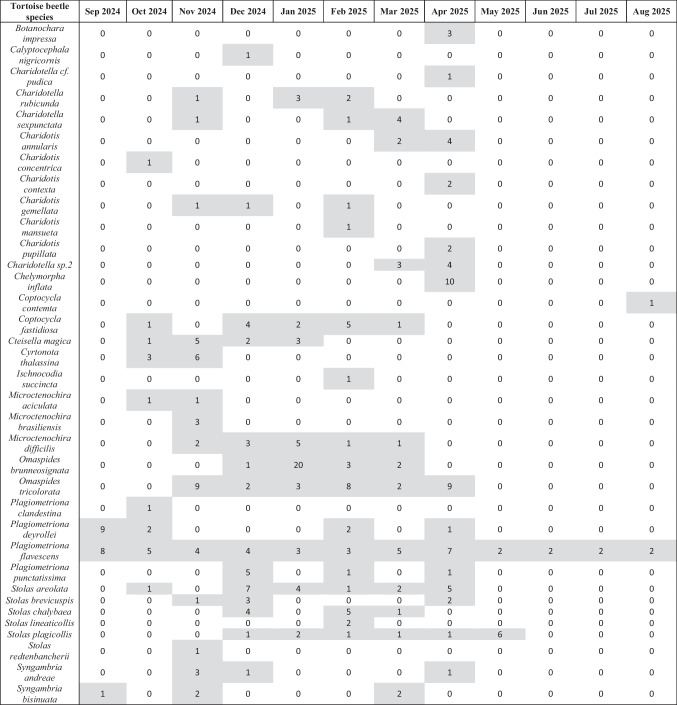
Abundance of adults of each tortoise beetle species (Cassidinae *s. str.*) found between September 2024 and August 2025 at Serra do Japi, Jundiaí, Brazil

**Table 3:** Circular statistics for the abundance and richness of adult tortoise beetles (Cassidinae *s. str.*) between September 2024 and August 2025 at Serra do Japi, Jundiaí, Brazil

	Abundance	Richness
**Number of observations**	277	95
**Mean vector (µ)**	22.129°	21.323°
**Mean group**	January	January
**Length of mean vector (*r*)**	0.437	0.413
**95% confidence interval (−/+) for µ**	11.765°	2.469°
	32.494°	40.177°
**Rayleigh test (*Z*)**	52.875	16.174
**Rayleigh test (*p*)**	< 0.001	< 0.001

**Fig. 9:**
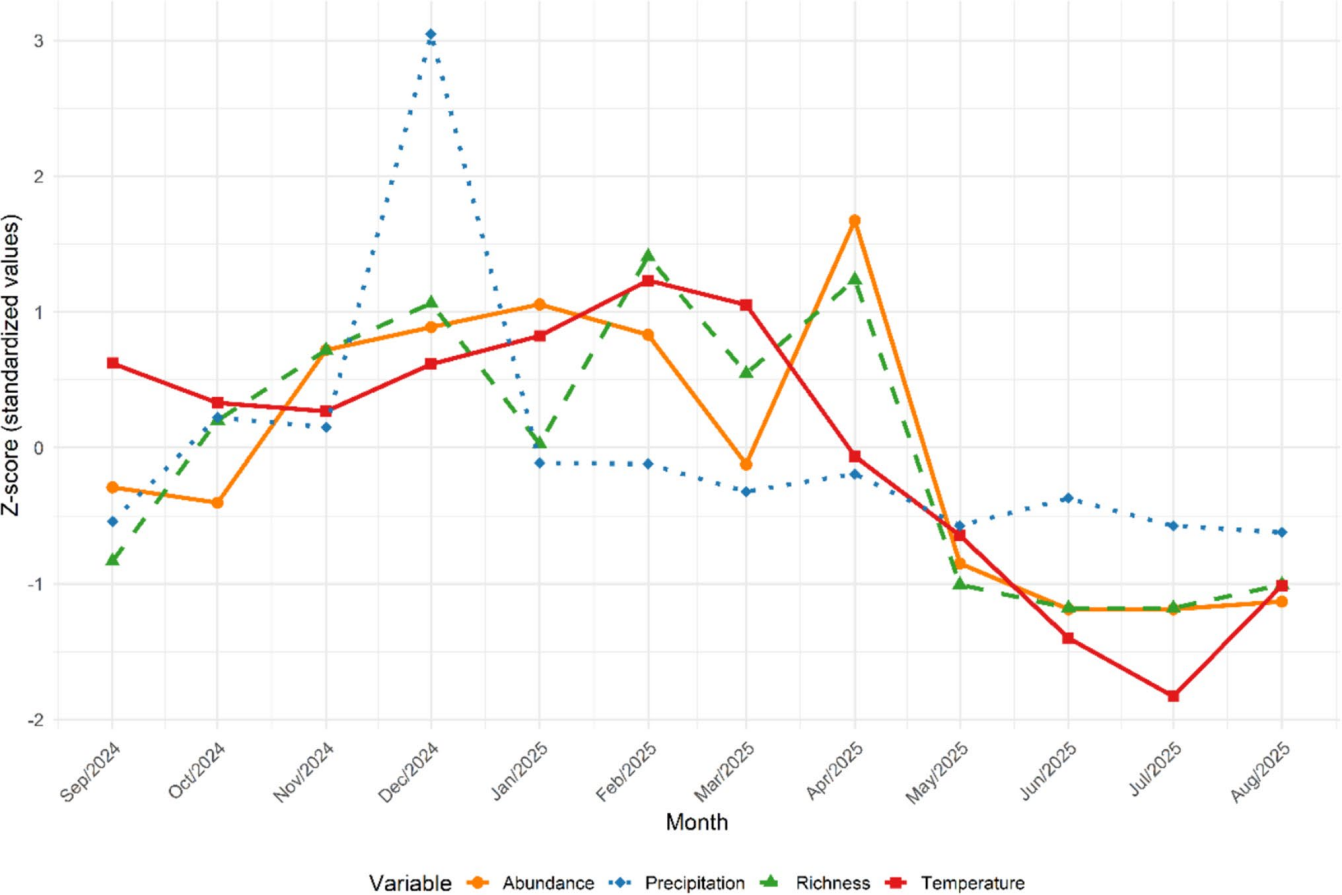
Standardized monthly variation of climatic (average temperature and accumulated precipitation) and assemblage (abundance and richness of adult Cassidinae *s. str.*) variables from September 2024 to August 2025 at Serra do Japi, Jundiaí, Brazil

**Fig. 10:**
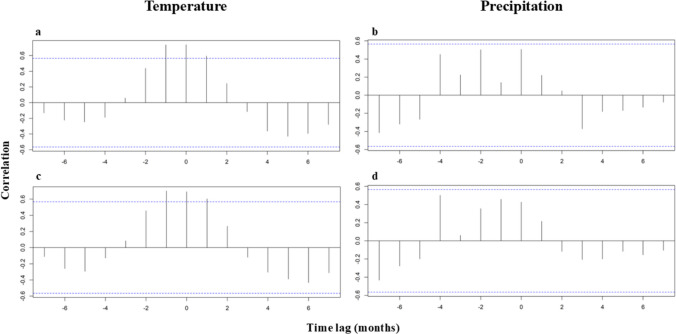
Cross-correlation between climatic variables and community metrics of adult *Cassidinae* s. str. at Serra do Japi, Jundiaí, Brazil (September 2024–August 2025). Upper panels (**a** and **b**) show species richness and lower panels (**c** and **d**) abundance. Left panels show correlations with average temperature and right panels with accumulated precipitation. Dashed horizontal lines indicate the 95% confidence interval; correlation peaks exceeding these limits are statistically significant. Negative lags indicate that climatic variables precede community responses

The cross-correlation function showed that temperature has a significantly positive linear influence on the abundance and richness (abundance: *β* = 4.147, *p* = 0.0125, *R*^2^ = 0.4277; richness: *β* = 1.4386, *p* = 0.00604, *R*^2^ = 0.5006) (Fig. 9). Temperature also shows a small delay of one month in its influence over the abundance and richness of the assemblage (Fig. 10 a and c). Precipitation, on the other hand, did not show a significant correlation with eighter assemblage variables for the studied period (abundance: *β* = 0.2052, *p* = 0.166, *R*^2^ = 0.1006; richness: *β* = 0.07922, *p* = 0.09252, *R*^2^ = 0.1827) (Fig. 10 b and d).

## Discussion

After 26 months of field work, 48 species of tortoise beetles (Cassidinae *s. str.*) were recorded associated with the edge vegetation of 3 trails and 3 roads Serra do Japi. Rarefaction curves and richness estimators indicate that our dataset is very representative for the studied assemblage but also estimates that more species are to be found in the area. Compared to the species list of Frieiro-Costa et al. (2012), 30 new species were recorded for the area, while 18 species previously reported were not detected. By joining both datasets, we present a list of 71 tortoise beetles’ species for Serra do Japi, with 11 new records for the state of São Paulo and 36 new host plant records. As predicted, our one-year analysis indicates that this tortoise beetle assemblage shows a clear seasonal pattern similar to subtropical areas. This temporal pattern was positively influenced by temperature variation as expected, but surprisingly not by precipitation.

### Tortoise beetle fauna and their host plants

Our final list presents a total of 71 species in 26 genera and six tribes of tortoise beetles recorded for Serra do Japi. This corresponds to 6.1% of the species registered for Brazil (Sekerka and Simões 2025). Among these, 28 species are endemic to the country, corresponding to 5% of the known endemic species. Groups found were very similar to that of other tortoise beetle surveys in Atlantic Forest regions, with Cassidini and Mesomphaliini as the main tribes (Simões and Monné 2011; Gomes et al. 2021). These tribes are the two richest tribes in Cassidinae *s.str.*, which probably explains their dominance in Neotropical community composition.

Our list also presents host plant records for 64 of the 71 species, with new records for 36 species, of these, 19 represent the first host plant records for the species. This represents a significant contribution to the knowledge of Neotropical cassidines host plant interactions, with 59 interactions confirmed at species level. Many studies on tortoise beetles treat their host plants as a secondary aspect and make little or no effort to identify them, which likely explains why most host plants are restricted to the family level when such information is available (Gomes et al. 2021). Species list for other areas of the Brazilian Atlantic Forest, for example, commonly do not include any information regarding the host plants of the assemblage being presented (Flinte et al. 2009; Simões and Monné 2011; Fernandes and Linzmeier 2012). Additionally, in our study, some beetles were found on plants from which they did not feed during rearing tests, highlighting the importance of feeding confirmation to validate host plant interactions. The total lack of host plant information or providing unreliable information based solely on field observations can both be harmful for future studies regarding tortoise beetles as these can either negatively interfere in sampling effort or result in misleading information on their host specificity.

Host plant families reported here are also present in the main registries (Flinte et al. 2008; Gomes et al. 2021), but we also included the habits of these plants. Vine species from Convolvulaceae, the most representative family, were the host plants of most species in the area. This seems to be common among Neotropical cassidines, reported on several other studies (Jolivet and Hawkeswood 1995; Chaboo 2007; Gomes et al. 2021). This strong association, however, needs to be taken with caution. Most studies, including this one, search for tortoise beetles on trail and road edges, forest borders, and open forest areas (Flinte et al. 2008, 2011; Gomes et al. 2021; Yang et al. 2024). These exposed environments favor forest border vegetation, such as vines, which may favor the occurrence of beetles associated with these plants. To reduce this bias, we sampled areas with different levels of disturbance and exposure including plants up to 3 m high, but we were still limited to trail/road edges where the highest plants were taller shrubs and young trees.

The species list presented here shows that Serra do Japi harbors a significant amount of Brazilian tortoise beetle fauna, with new host plant records and 11 new occurrences records for the state of São Paulo, one of them being a new record for Brazil. These are great indicators of the importance of Serra do Japi as a conservation unit, harboring a great number of species that are considerably rare and overall understudied in terms of their ecological aspects. Unfortunately, the region is also highly threatened by anthropic activity such as habitat loss since it is surrounded by big urban centers and three important highways (Anhanguera, Bandeirantes and Castelo Branco). The recorded assemblage of species is composed of specialized feeders mostly associated with forest edge vegetation, such as vines and shrubs, that are commonly removed and/or suppressed to clear trails, roads, and highways. During our sampling period, even when vegetation was allowed to grow back, it was very common for such areas to be dominated by fast-growing invasive species that outcompeted the native ones. Increasingly anthropic activity in the region can intensify this, heavily impacting low-abundance insect feeders that depend on forest edge vegetation for their survival. This shows how vulnerable this conservation unit and the species harbored by it are, highlighting the importance of biodiversity research to be developed in the area. Additionally, we believe that trail and road management inside the conservation unit should be better planned to avoid completely removing edge vegetation as much as possible, minimizing the impact on Cassidinae *s. str.* populations.

### Seasonality influenced by climatic factors

Our 1-year analysis indicates that the tortoise beetle community of Serra do Japi shows a clear seasonal pattern. This pattern tracks local seasonal changes, with highest abundance and richness during the warm and rainy months, while most species overwinter in diapause (Wolda 1988; Medeiros and Vasconcellos-Neto 1994). Even the only species recorded throughout the year, *P. flavescens*, showed a significant decrease in abundance during the cold and dry season (Table 2). This is in contrast to other tropical areas of Brazil where, according to Nogueira-de-Sá et al. (2004), Cassidinae *s. str.* tend to occur throughout the year and are little influenced by climatic factors. Nonetheless, the altitude of the conservation unit gives it climatic variation more similar to subtropical areas, sometimes referred to as “high-altitude tropical climate,” which might explain the observed pattern of this insect assemblage (Flinte et al. 2011). Additionally, although the conservation unit was treated as a single location, Serra do Japi is a mountain range. As shown by Flinte et al. (2011), microclimatic factors and host plant availability may vary across altitudinal gradients and change differently throughout the year, potentially leading to distinct temporal patterns for the same tortoise beetle assemblage. Given this, the altitudinal gradient of Serra do Japi warrants further investigation.

Consistent with the general pattern reported for Neotropical tortoise beetles, immatures were only found during the hot and rainy season, specifically between December and April, which seems to be the reproductive period of the group. *Plagiometriona deyrollei* was the only species for which immatures could already be found in October. Since this species did not share its host plant with any other species, which could cause such a difference to avoid competition for example, it is unclear why it deviated from the rest of the assemblage.

As expected, there was significant influence of temperature in the temporal variation of this species assemblage, in which increased temperatures lead to an increase in abundance and richness of adult tortoise beetles. Insects in general show a positive relationship with temperature since warm weather tends to favor their metabolism, favoring the abundance of the different species (Messenger 1959; Silva et al. 2011; Sable and Rana 2016). Additionally, the cross-correlation analysis revealed a response lag, with abundance and richness in a given month being influenced by the temperatures of both the current month and the preceding month. This pattern is evident in April, for instance, when temperatures had already begun to decline, yet a peak in abundance and richness was still observed (Fig. 9). This delayed response likely reflects the presence of immatures that thrived during the previous month when temperatures were higher, reaching adult stage at the end of the season.

Surprisingly, precipitation did not show a significant correlation to the assemblage’s temporal variation. Nonetheless, immature stages are more sensitive to abiotic changes than adults, which limits our conclusions on their relative importance over these beetles’ assemblage since richness and abundance data were quantified based on adults only (Flinte et al. 2011). For example, while adult beetles peaked from October to April, immatures of most species were only seen after December, which was the month with the higher precipitation in the period (personal observations). Rainfall is commonly associated with a higher rate of plant growth, which can favor reproduction in herbivorous insects due to increased food-source availability (Morais and Diniz 2004; Oliveira et al. (2021). Therefore, precipitation might influence temporal patterns when immatures are accounted for (Flinte et al. 2011). However, quantitative data is required to confirm this.

Because we compare temporal variation with only two climatic variables over a single year, these results should be interpreted with caution. Irregular rainfall patterns or atypical precipitation and temperature levels may have influenced the observed patterns. Surveys throughout multiple years and including different life stages rather than only adults can provide more robust insights into these dynamics. Given the host specificity of tortoise beetles, host plant availability and phenology might also play an important role in the temporal distribution of these beetles, perhaps even more than climatic factors. Thus, data on the phenological patterns and temporal distribution of host plants should also be considered in future studies to better understand Cassidinae *s. str.* dynamics.

### Sampling effort

Between March 2023 and September 2025, we sampled 48 species at Serra do Japi, a richness quantitively similar to that reported by Frieiro-Costa et al. (2012) for the same area between 1988 and 1995 (41 confirmed species). Given that the list in Frieiro-Costa et al. (2012) was based on occasional encounters with the species in the area, we expected that a systematic sampling effort would be enough to envelop all species previously recorded and record new ones. Nonetheless, despite the comparable species totals, only 23 species were shared between the two sampling periods, which are separated by approximately 25 years. During the interval between surveys, several factors may have impacted the composition of the cassidine assemblage. Wildfires, such as the event that burned approximately 12 ha of the area in 2018, and changes in the routine of periodic trail-management that happen every 4 years, represent just two possible examples of local disturbances. Events like these can drastically decrease the population of plants associated with roads and trails’ edges or even change the vegetation composition of these environments. Given the host specificity of tortoise beetles, these changes can affect the Cassidinae *s. str.* species found in the area by limiting host plant availability and increasing interspecific competition, potentially shifting the composition of the tortoise beetle assemblage. These could help explain the marked compositional difference of the sampled assemblage between the two surveys. Unfortunately, given that Frieiro Costa et al. (2012) is solely a list of recorded species, we lack abundance data from previous surveys, limiting our capability to discuss these differences any further.

The multinomial model results indicate that our sampling effort covers a significant portion of the species assemblage expected to be found in the area (SC = 0.9695). The model estimated that even with a near doubling of the sampling effort (from 393 to 700 specimens), no more than 15 additional species would be expected to be recorded. The other richness estimators followed a similar pattern, showing expected richness values of 61.2 species (Chao1) and 58.6 species (ACE). These results indicate a tendency toward stabilization of the species accumulation curve, making our data set relevant to study the tortoise beetle assemblage associated with edge vegetation, although additional rare species may still be detected with increased sampling effort. Even though they are a highly diverse group, most tortoise beetles are generally considered rare species, with very low densities in nature, which increases the difficulty of sampling all expected species (Windsor et al. 1992; Jolivet and Hawkeswood 1995; Flinte et al. 2008, 2015; Frieiro-Costa et al. 2012). For example, among 1590 individuals of Cassidinae sampled by Liu et al. (2019), almost 90% of them were hispines, with only around 10% being tortoise beetles, showing their rarity when compared with their closely related group. Flinte et al. (2015) also estimated that more or less one individual of *Omaspides trichroa* per host plant is expected on periods of high abundance. Given this species presents maternal care, which can favor the survival of immatures and increase the abundance of adults, even lower abundances are expected for species without maternal care. Our data supports this by showing that two of the species with the highest total abundance between 2024 and 2025, *O. tricolorata* and *O. brunneosignata*, are also the ones that present maternal care.

Active search is a time-consuming and labor-intensive method to sample insects, especially for low abundance groups such as tortoise beetles since hours in the field are often spent only to find a few specimens (Windsor et al. 1992; Frieiro-Costa et al. 2012; Flinte et al. 2015). Passive collection (Malaise, pitfall, and light traps) is unfortunately not effective for sampling this group due to their limited mobility, and such methods do not allow the detection of their host plants (Jolivet & Hawkeswood 1995; Flinte et al. 2009). Therefore, even with its difficulties, active search remains the best way to document these tortoise beetles’ interactions and distribution. Additionally, information about their host plants may reduce the time search for cassidines, minimizing one of the negative points of this collection method (Fernandes and Linzmeier 2012; Gomes et al. 2021).

## Supplementary Information

Below is the link to the electronic supplementary material.

ESM 1 (XLSX 17.9 KB)

## Data Availability

All data generated or analyzed during this study are included in this published article and its Supplementary Information files. The R scripts used for data analyses are available from the corresponding author upon reasonable request.
